# A Novel Mouse Model of Aminoglycoside-Induced Hyperacusis and Tinnitus

**DOI:** 10.3389/fnins.2020.561185

**Published:** 2020-09-18

**Authors:** Ryan J. Longenecker, Rende Gu, Jennifer Homan, Jonathan Kil

**Affiliations:** Sound Pharmaceuticals, Inc., Seattle, WA, United States

**Keywords:** hearing loss, aminoglycoside, amikacin, hyperacusis, tinnitus, ebselen

## Abstract

Aminoglycosides (AG) such as amikacin are commonly used in cystic fibrosis patients with opportunistic pulmonary infections including multi-drug resistant mycobacterium tuberculous and non-tuberculous mycobacterium. Unfortunately, this class of drugs is known to cause peripheral damage to the cochlea leading to hearing loss that can fluctuate and become permanent over time or multiple exposures. However, whether amikacin can lead to central auditory dysfunction like hyperacusis (increased sensitivity to sound) or tinnitus (perception of sound in the absence of acoustic stimulation) is not well-described in the literature. Thus, an animal model needs to be developed that documents these side effects in order to develop therapeutic solutions to reduce AG-induced auditory dysfunction. Here we present pioneer work in mice which demonstrates that amikacin can lead to fluctuating behavioral evidence of hyperacusis and tinnitus as assessed by the acoustic startle reflex. Additionally, electrophysiological assessments of hearing via auditory brainstem response demonstrate increased central activity in the auditory brainstem. These data together suggest that peripheral AG-induced dysfunction can lead to central hyperactivity and possible behavioral manifestations of hyperacusis and tinnitus. Importantly, we demonstrate that ebselen, a novel investigational drug that acts as both an antioxidant and anti-inflammatory, can mitigate AG-induced hyperacusis.

## Introduction

Aminoglycoside (AG) antibiotics are the most prevalent treatment option for CF and other life-threatening gram-negative bacterial infections (Flume et al., 2009; Drusano and Louie, 2011). However, cautionary results have shown that extended treatment with AGs such as tobramycin or amikacin can cause permanent hearing loss (Jiang et al., 2017). Such findings are now becoming more prevalent in clinical literature suggesting that AG-induced hearing loss is a serious concern for patients requiring treatments throughout life (American Speech-Language-Hearing Association [ASHA], 1994; Garinis et al., 2017). For these reasons, preclinical models are needed to understand the nature of AG-induced cochleotoxicity in order to develop solutions to prevent clinical auditory loss and dysfunction.

Animal models have demonstrated varying degrees of AG-induced cochlear hair cell damage and subsequent hearing loss (Huth et al., 2011; Ogier et al., 2020). However, emerging evidence suggests that SGNs, and their specialized ribbon synapses may also be damaged by AGs (Sone et al., 1998; Pauna et al., 2017; Hong et al., 2018). Such peripheral pathology evidenced in models of noise-induced and age-related hearing loss (Kujawa and Liberman, 2015), has been shown to contribute to central maladaptive plasticity (Gold and Bajo, 2014; Eggermont, 2017). Tinnitus, or ringing in the ears, and hyperacusis, a heightened sensitivity to sound, are both thought to be symptoms of this central plasticity (Knipper et al., 2013). However, it is not known whether AGs can cause these complex auditory dysfunctions. Mechanistically, it is likely that inflammation plays a substantial role in AG-induced cochleotoxicity and auditory dysfunction (Jiang et al., 2017; Wood and Zuo, 2017). AGs have been shown to potentiate hearing loss and cochlear damage in animal models of systemic inflammation (Hirose et al., 2014; Koo et al., 2015). It has also been shown that AG’s cause neural inflammation mediated by NMDA receptors (Grill and Maganti, 2011), leading to neuromuscular diseases or brain lesions. However, it is unknown whether AG-induced inflammation can result in hyperacusis and tinnitus.

SPI-1005 (ebselen), a glutathione peroxidase mimic and inducer, has novel anti-inflammatory activity and has been shown to protect hair cells from various insults including noise (Kil et al., 2007, 2017), cisplatin (Lynch et al., 2005), and aminoglycosides (Gu et al., 2020). It is not yet known if the antioxidant and/or anti-inflammatory properties of ebselen can prevent/treat the central components of amikacin-induced cochleotoxic changes (Kalinec et al., 2017). However, strong support for the central effect of ebselen has been shown in disease models of inflammation such as Alzheimer’s (Martini et al., 2019), Parkinson’s (Moussaoui et al., 2000), bipolar disorder (Singh et al., 2013), and schizophrenia (Cabungcal et al., 2014). It is possible that ebselen may also alleviate common centrally based otolaryngologic diseases such as AG-induced hyperacusis or tinnitus. Indeed, recent studies have shown that reducing inflammation caused by auditory insults can ameliorate behavioral signs of tinnitus in animals (Wang et al., 2019).

The goals for this study were to develop an amikacin-induced auditory loss and dysfunction mouse model. First, we investigated if a clinically relevant dosing schedule of amikacin led to hearing loss (Ogier et al., 2020). Hearing functionality was assessed via changes in ABR thresholds and ABR wave amplitudes (Gu et al., 2012; Lowe and Walton, 2015). Second, we determined if amikacin could lead to hyperacusis (Pienkowski et al., 2014) or tinnitus (Galazyuk and Hébert, 2015) using the ASR. Third, hair cells, SGNs, and ribbon synapse loss was observed using cochlea whole mount and cross section histology. Finally, we investigated whether ebselen’s anti-inflammatory properties were able to mitigate amikacin-induced auditory dysfunction for any of the aforementioned assays.

## Materials and Methods

### Subjects

**FIGURE 1:**
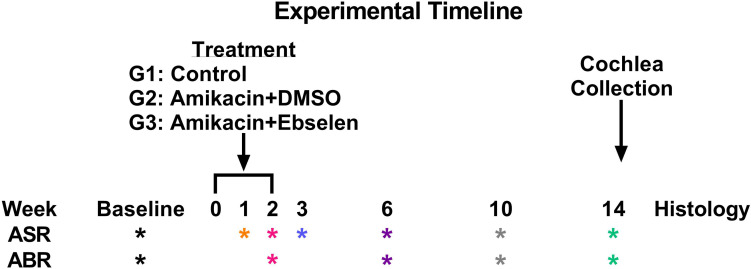
Experimental timeline. ASR and ABR tests (^∗^ symbol) are color coded to specific weeks the tests occurred after the start of treatment. This color code is maintained throughout the manuscript. Treatment regimens were carried out from week 0 to 2 for groups (G) 2 and 3, while group 1 did not receive any treatment. Animals were sacrificed after week 14 tests for cochlear histology.

A total of 30 male/female CBA/Ca mice 3 months of age (at the start of experiments) were used in this study. Seventeen mice were included in all behavioral and electrophysiological studies (and some in histological studies), while a subset of mice was used for only ABR and histological studies. Mice were born in house from parents obtained from Jackson Laboratories. Mice were housed 3–4 to a cage within a colony room with a 12-h light–dark cycle at 23°C. Hearing and behavior was tracked longitudinally for each animal in a repeated measures design and animals were sacrificed for cochlear histology 14 weeks after the start of experimentation (Figure 1).

### Drug Formulation, Dosing, and Schedule

Stock ebselen powder was dissolved in pure DMSO at 20 mg/ml and stored at minus 20°C. Ebselen (20 mg/ml in DMSO) at 20 mg/kg body weight was diluted in fresh 0.5 ml sterile saline. Mice were divided into three groups: Group 1 (*n* = 6) served as a control and did not receive amikacin or ebselen. Group 2 (*n* = 13) received the vehicle (DMSO at equal volume to G1 on a per weight basis) i.p. and amikacin s.c. 30 min later. Group 3 (*n* = 11) received ebselen i.p. and amikacin at 500 mg/kg body weight s.c. 30 min later. The daily dosing for Groups 2 and 3 was identical and was continued for 14 days. During the dosing period, the health and condition of animals were monitored by body weight, which is known to decrease during AG treatments, and daily behavioral observation.

### Auditory Brainstem Response

Mice were anesthetized with isoflurane. Basal body temperature was maintained using a Gaymar T-pump warming pad set to 37°C and the animals’ health was monitored by observation of respiration and circulation. Each ear was otoscopically inspected prior to insertion of ear tips (Nicolet Biomedical, Inc.) for sound delivery. Monaural closed field ABRs (Intelligent Hearing Systems) were collected before (baseline), as well as at weeks 2, 6, 10, and 14 from the start of AG treatment (Figure 1). Subdermal platinum needle electrodes (Grass Telefactor, Inc.) were placed with the active electrode at the vertex and the reference electrode to the test ear, and the ground to the contralateral ear. Each ear was tested independently. Stimuli consisted of pure tone pips (5 ms duration, rectangular envelope) at 4, 8, 16, and 32 kHz presented for 800 repetitions (19.3 r/s) at sound levels from 60 to 0 dB SPL (initially 20 dB steps until near threshold, then 5 dB steps) calibrated with a 0.25 inch microphone (Brüel and Kjaer, 4939). Thresholds were measured in 5 dB increments and defined visually by the presence of the most robust peak (I or III) that was reliable within 0.1 ms. Thresholds were analyzed by a scientist blind to treatment and isolated from data collection.

### Behavioral Assessments of Hyperacusis, Tinnitus

#### Acoustic Startle Hardware/Software

Startle Reflex Hardware was purchased from Proxima Centauri Technologies (Julian, CA, United States). Each startle cabinet was lined with Sonex anechoic foam to minimize sound reflection and wave canceling sound echoes (Longenecker and Galazyuk, 2012). Sound levels from each cabinet’s speakers was calibrated with a 0.5-inch microphone (Brüel and Kjaer 4939). Startle Waveforms were recorded using load-cell platforms and calibrated with 100 g weights. Offline data processing with code written in visual basic was used to evaluate whether each trial was a startle or non-startle via template matching and startle magnitude data was converted from force to CMD (Grimsley et al., 2015). Only legitimate startles were included and used in the final data analyses (Longenecker et al., 2018).

#### Input/Output Functions for Hyperacusis Assessments

Startle stimuli were pseudorandomly presented between 60- and 100-dB SPL in 5 dB steps. Intertrial intervals were randomized between 4 and 6 s. Each input/output (I/O) session lasted roughly 12 min and consisted of 135 total trials in which each startle intensity was presented 15 times. I/O assessments were collected before (baseline), as well as at weeks 1, 2, 3, 6, 10, and 14 from the start of AG treatment (Figure 1).

#### GPIAS for Tinnitus Assessment

Gap prepulse inhibition of the ASR was used to assess behavioral evidence of tinnitus (Longenecker and Galazyuk, 2011, 2016). The ability of mice to detect a gap of silence preceding a startle stimulus was determined by comparing the startle magnitude in response to a startle stimulus (white noise; 100 dB SPL) presented alone (SO) and a startle stimulus paired with a preceding (100 ms before) gap (20 ms long) of silence (GAP). Both trials were presented in a continuous narrowband noise carrier presented at five different frequencies (4, 8, 12.5, 16, 20 kHz) at a constant intensity of 65 dB SPL. Additionally, 15 startles presented in silence were used to monitor startle habituation. Intertrial intervals were randomized between 4 and 6 s.

A testing session was comprised of 15 blocks comprising 150 trials, lasting roughly 15 min. A block was defined by 10 trials containing five pseudorandom SO and GAP trials presented in a uniform carrier frequency. Throughout the session, each carrier frequency block was represented three times for a total of 45 trials. On each testing day, 3 GPIAS sessions were run on each mouse lasting roughly 45 min. The best performance ratio was used to determine an individual animal’s daily gap detection performance (Longenecker et al., 2018). GPIAS assessments were collected before (baseline), as well as at weeks 1, 2, 3, 6, 10, and 14 from the start of AG treatment (Figure 1).

### Cochlear Histology

#### Whole Mount Epifluorescence

Following the final ABR and behavioral assessments (Figure 1), mice (∼6–7 months old) were sacrificed with CO_2_. Cochlea were collected and fixed in 4% PFA overnight. A subset of left cochlea from three different groups (amikacin/DMSO *n* = 5; amikacin/ebselen *n* = 5; untreated control *n* = 3) were processed for whole mount immunostaining. After the bony wall was removed carefully, the intact membranous cochlea was isolated from the modiolus. After decalcification in 0.5M EDTA for 1 h, the membranous cochlea was permeabilized and blocked in 0.2% Triton X-100, 1% BSA, and 5% donkey serum in PBS. When assessing damage done to the organ of Corti, the tissue was incubated with two primary antibodies: Rabbit anti-Calretinin (1:200 dilution) and Goat anti-Prestin-N20 (1:200 dilution) overnight at 4°C, rinsed in PBS, and incubated with two secondary antibodies: Alexa Fluor 594 Donkey anti-Rabbit IgG (1:500 dilution), Alexa Fluor 488 Donkey anti-Goat IgG (1:500 dilution) for 2 h at room temperature. For the ribbon synapse observation, tissue was incubated with two primary antibodies: Rabbit anti-GluR2 (1:500 dilution) and Mouse anti-CtBP2 (1:500 dilution) overnight at 4°C, rinsed in PBS, and incubated with two secondary antibodies: Alexa Fluor 594 Donkey anti-Rabbit IgG (1:500 dilution), Alexa Fluor 488 Donkey anti-mouse IgG (1:500 dilution) for 2 h at room temperature. The membranous cochlea was cut at the apical turn and the basal turn, then further dissected, embedded in mounting media with DAPI. Samples were examined via an epi-fluorescent microscope (Nikon Eclipse Ti) and images were captured via a CCD camera (Hamamatsu C11440).

#### Cross Section Light and DIC Microscopy

For paraffin embedding, the right cochlea from the amikacin/DMSO group (*n* = 8) and amikacin/ebselen group (*n* = 9) were decalcified in 0.5M EDTA for 5 days, and then prepared for sectioning on a microtome. The mid-modiolar sections were cut at 7 μm thickness, stained with 1% Toluidine blue and examined under light and DIC microscopy (≥ 7 sections per cochlea).

### Data Analysis

GraphPad Prism 8 was used for statistical analysis. One-way and two-way ANOVAs were used in data sets with normally distributed and equal sample sizes. Mixed models analyzed data that did not meet these assumptions. Sidak’s multiple comparison tests were used to discover individual differences at specific timepoints in the *in vivo* dosing study. Fisher’s Exact Test with relative risk assessments were used to analyze clinically relevant ABR threshold changes (Table 1). ^∗^*p* ≤ 0.05; ^∗∗^*p* ≤ 0.01; ^∗∗∗^*p* ≤ 0.001.

**TABLE 1:** Clinically relevant change (CRC) for ABR threshold shifts at 4, 8, 16, and 32 kHz between testing groups 2 and 3.

Week	Amikacin + DMSO (*n* = ears)	Amikacin + Ebselen (*n* = ears)	*p*-Value	Effect size
2	18 (22)	10 (20)	0.664	1.82
6	36 (22)	25 (20)	0.514	1.46
10	18 (22)	0 (20)	0.109	∞
14	0 (16)	0 (20)	>0.999	NA

*(1) Criterion for CRC: A ≥ 20 dB shift at one frequency. (2) A ≥ 15 dB shift at two adjacent frequencies. (3) A ≥ 10 dB shift at three adjacent frequencies. Each ear was analyzed independently. The percentage of ears which met the ABR threshold shift criteria was calculated for each time point (weeks 2, 6, 10, 14). Two-sided Fisher’s exact tests were used statistically evaluate differences between testing groups. The effect size represents relative risk (Koopman asymptotic score).*

To develop clinically relevant ABR threshold shift criterion, we followed ASHA guidelines for ototoxic change using pure tone audiometry (American Speech-Language-Hearing Association [ASHA], 1994; Gu et al., 2020). Here, we identified ototoxic change using the following three criteria: (1) A ≥ 10 dB shift at three adjacent tested frequencies (4, 8, 16, 32 kHz). (2) A ≥ 15 dB shift at two adjacent tested frequencies. (3) A ≥ 20 dB shift at any one tested frequency. Each ear was analyzed independently for each animal tested. The percentage of ears which met the threshold shift criteria was calculated for each time point (weeks 2, 6, 10, 14; Figure 1 and Table 1).

## Results

### Amikacin Causes Mild Fluctuating Threshold Shifts in the Absence of Obvious Cochlear Damage

**FIGURE 2:**
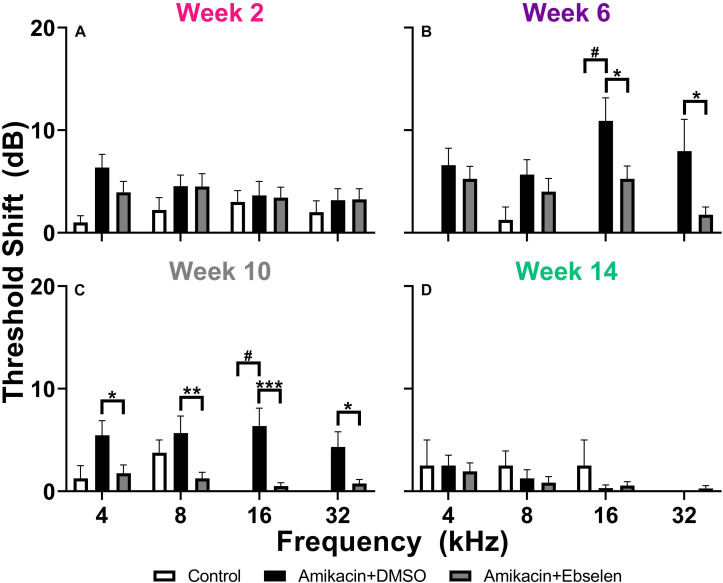
Averaged ABR threshold shifts comparing testing groups at different epochs (**A:** week 2, **B:** week 6, **C:** week 10, **D:** week 14) after the start of treatment. ABRs were collected for 4, 8, 16, and 32 kHz. Shifts represent the specific timepoint minus the baseline ABR value for each group. Data are represented by threshold shift means and standard errors. *Post hoc* tests determined significant differences between testing groups, which are indicated as follows: #, between control and DMSO treated animals; # (gray), between control and ebselen treated animals; ^∗^, between DMSO and ebselen treated animals.

To examine the effects of amikacin on hearing sensitivity, we documented ABR thresholds up to 14 weeks from the start of AG treatment (Figure 1). Following a standard 14-day amikacin regimen (2 weeks), ABR thresholds were only slightly elevated (∼5 dB) from baseline levels and no significant differences were observed between groups [*F*(2,193) = 3.039, *p* = 0.0502] (Figure 2A). However, threshold shifts for amikacin treated animals increased at weeks 6 [*F*(2,171) = 8.207, *p* = 0.004] and 10 [*F*(2,172) = 14.48, *p* < 0.0001] (Figures 2B,C) compared to controls, and decreased to near-baseline levels at week 14 [*F*(2,140) = 0.8673, *p* = 0.4223] (Figure 2D). *Post hoc* tests revealed significance between control and DMSO treated animals at 16 kHz (*p* = 0.0131), as well as for DMSO and ebselen treated groups at 16 kHz (*p* = 0.0256) and 32 kHz (*p* = 0.0131) at 6 weeks. At 10 weeks, significance was found between control and DMSO treated animals at 16 kHz (*p* = 0.0344) as well as for DMSO and ebselen groups at 4 kHz (*p* = 0.0304), 8 kHz (*p* = 0.0098), 16 kHz (*p* = 0.0007), and 32 kHz (*p* = 0.0369). In a separate analysis, we used our recently developed clinically relevant changes criteria to determine cochleotoxicity for each ear (per animal) using ABR threshold data (Gu et al., 2020; Table 1). Using these criteria, there were only a small percentage of ears that had clinically relevant hearing loss. Fishers LSD test did not reveal any significant differences between treatment groups at any epoch (Table 1). These findings taken together show that amikacin given at this dose in mice caused a mild fluctuating hearing loss which recovered by 14 weeks after the start of treatment and was mitigated by ebselen co-treatment.

**FIGURE 3:**
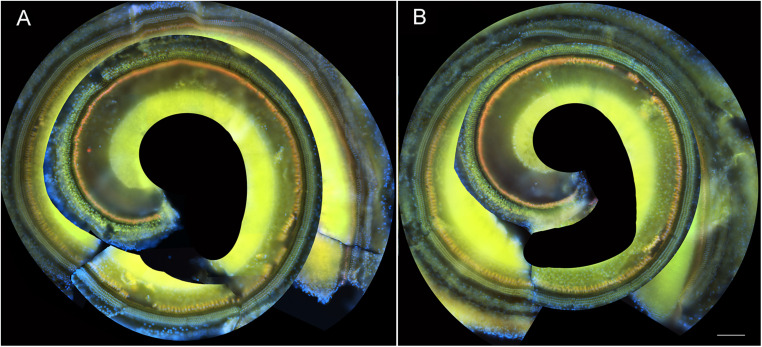
Representative whole mount cochlear images displaying cochlear hair cells for both treatment groups (**A:** amikacin and **B:** amikacin/ebselen). No observable evidence of amikacin-induced OHC (stained with anti-Prestin antibody, green) or IHC (stained with anti-Calretinin antibody, red) loss. Scale bar = 100 mm.

**FIGURE 4:**
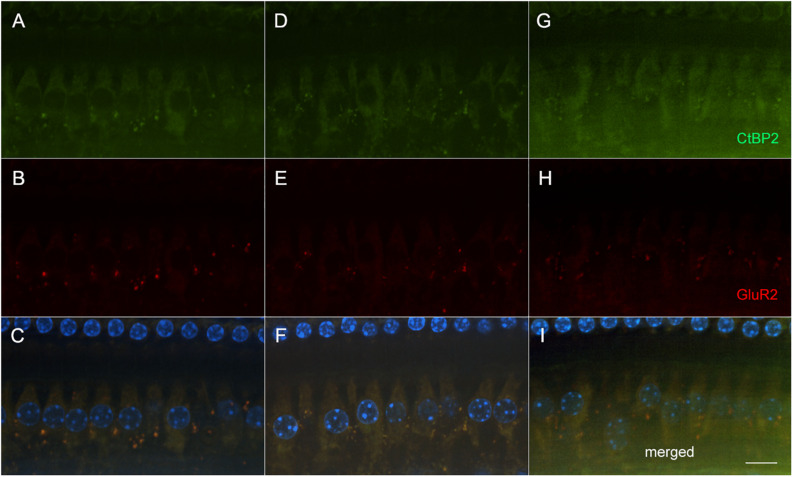
Representative micrographs showing pre and post-synaptic densities in the 20 kHz region in three groups following AG treatment (**A–C**: untreated controls**, D–F**: amikacin + DMSO, and **G–I**: amikacin + ebselen). No observable differences of immunolabeled presynaptic marker CtBP2 (green; **A,D,G**), post-synaptic marker GluR2 (red; **B,E,H**), or the merged images which include hair cell nucleus marker DAPI (blue; **C,F,I**) were seen between groups. Scale bar = 10 mm.

To determine if these amikacin-induced threshold shifts were directly caused by loss of cochlear hair cells or SGNs, we examined cochlea from animals which were sacrificed after the final behavioral testing 14 weeks after the start of treatment (Figure 1). Immuno-florescent staining of hair cells demonstrated no observable inner or outer hair cell loss throughout the cochlea in either amikacin/DMSO or amikacin/ebselen treatment groups (Figures 3A,B). When observing pre- and post-synaptic hair cell densities, differences in the density of ribbon synapses between untreated control animals (Figures 4A–C), animals treated with amikacin/DMSO (Figures 4D–F), or animals treated with amikacin/ebselen (Figures 4G–I) were not obvious. Mid-modiolar serial cross sections confirmed the absence of damage to hair cells and SGNs (Supplementary Figure 1). These results together suggest that this dose and dose schedule (500 mg/kg for 14 days) of amikacin may not cause permanent damage to the mouse organ of Corti.

### Amikacin May Induce Hyperactivity and Behavioral Evidence of Hyperacusis and Tinnitus

**FIGURE 5:**
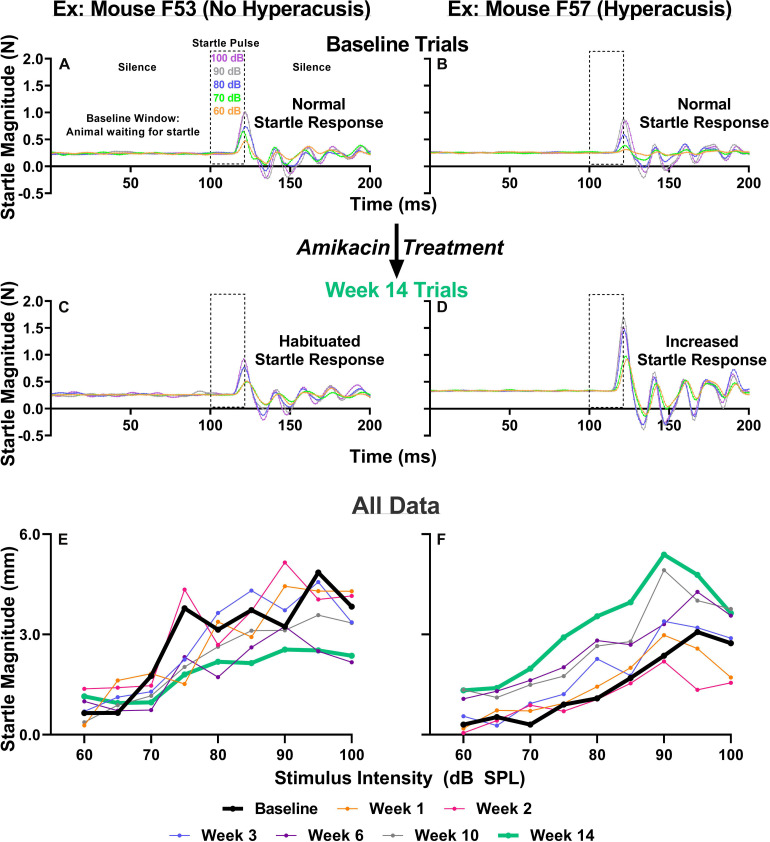
**(A–D)** IO paradigm, including the stimuli used and the corresponding animal reactions. Representative baseline IO trial data (five trials; one trial each for startle pulses presented at 60, 70, 80, 90, and 100 dB SPL) from two representative animals **(A,B)**. Representative 14-week post amikacin treatment IO trial data from an animal who did not develop hyperacusis **(C)** and from an animal who did develop hyperacusis **(D)**. The resulting startle response wave forms are color-coded to the level of startle stimuli presented. **(E,F)** All IO Startle stimulus/response functions recorded from the two representative mice (from **A–D**). Startle response magnitude means are plotted as a function of stimulus intensity (60 to 100 dB SPL in 5 dB steps) and colored-coded based on specific testing epochs {baseline, 1, 2, 3, 6, 10, and 14 weeks}. Notice the differences between the bolded black lines (baseline; **A,B**) and bolded green lines (week 14; **C,D**) from the animal without hyperacusis **(C)**, compared to the animal with hyperacusis **(D)**. Startle Magnitude recorded via force (N) in **(A–D)** was converted to CMD (mm) in **(E,F)**.

**FIGURE 6:**
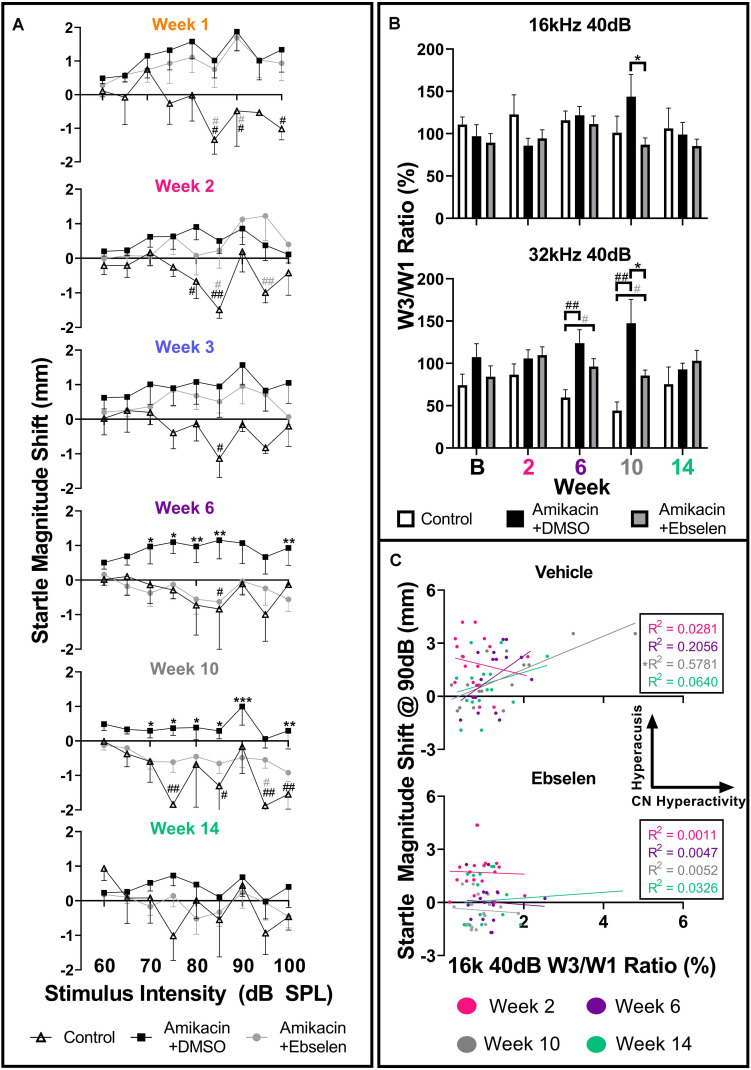
Evaluation and comparison of behavioral evidence of hyperacusis and electrophysiological evidence of increased central gain over time between treatment groups. **(A)** Averaged IO stimulus/response curves at multiple timepoints (time from the start of treatment {1, 2, 3, 6, 10, 14 weeks} – baseline) showing changes in startle response magnitude CMD (mm) as a function of stimulus intensity (60 to 100 dB SPL). The value 0 on the y-axis represents no change from baseline startle. Note the difference in startle magnitude change between control and amikacin treated mice until week 14. Data show significant effects of ebselen treatment at week 6 and 10 (see Supplementary Table 1 for significance details). **(B)** Averaged ABR wave III/I amplitude ratios from baseline and multiple timepoints after treatment. **(C)** A similar linear regression shows the correlation between startle at 90 dB SPL (from **A**) and 40 dB SPL W3/W1 amplitude ratios (from **B**) increased from week 2 to week 10 for the vehicle group but not the ebselen group. In **(A,B)**, data are represented by means and standard errors. *Post hoc* tests determined significant differences between testing groups, which are indicated as follows: #, between control and DMSO treated animals; # (gray), between control and ebselen treated animals; ^∗^, between DMSO and ebselen treated animals.

Previous studies have suggested that behavioral evidence of hyperacusis can be observed if an animal’s startle response magnitude increases from baseline levels following an insult to the auditory system. To test for this possibility, we conducted ASR input/output tests which examine an animal’s startle response as a function of sound intensity at weeks 1, 2, 3, 6, 10, and 14 after the start of treatment (Figure 1). We found that amikacin treatment led to substantial and significant increases in startle response magnitude both in individual animals and overall group averages (Figures 5B,D,F, 6A). Amikacin treatment led to dramatically increased startle magnitudes (individual change from baseline and as compared to controls) beginning in the first week and extending throughout the 14-week testing and follow-up period (Figure 6A). Two-way ANOVAs demonstrated significance for treatment at week 1 [*F*(2,144) = 8.728, *p* = 0.0003], week 2 [*F*(2,162) = 8.399, *p* = 0.0003], week 3 [*F*(2,153) = 8.495, *p* = 0.0003], week 6 [*F*(2,144) = 22.16, *p* < 0.0001], week 10 [*F*(2,144) = 30.44, *p* < 0.0001], and week 14 [*F*(2,144) = 3.669, *p* = 0.0279]. *Post hoc* analyses revealed many significant differences between groups at specific epochs and stimulus intensities (see Supplementary Table 1). Interestingly, mice co-treated with ebselen demonstrated a reduction from elevated startle levels by week 6, while the DMSO group did not recover to near-baseline levels until week 14. We found that 14 out of 17 mice given amikacin developed behavioral evidence of hyperacusis at a minimum of one follow-up timepoint, with most mice showing enhanced startle responses at multiple timepoints, see mouse #F57 (Figure 5F). The remaining three AG-treated mice demonstrated decreased startle responses over time (individual example in Figures 5A,C,E), a pattern demonstrated by control animals (Figure 6A), which may represent a habituation to the startle.

To determine if these amikacin-induced behavioral abnormalities were correlated to electrophysiological increases in central gain, we assessed ABR wave amplitudes. When looking at raw ABR amplitudes between amikacin treated mice as a function of frequency and time, only slight, non-significant differences were found between the DMSO and ebselen groups (Supplementary Figure 2). Wave one amplitudes showed a general reduction until 10 weeks following treatment, and wave three amplitudes increased slightly over the same period (Supplementary Figure 2). Using the ABR wave III over wave I ratio for 40 dB SPL stimuli for each ear at each epoch, we determined that this ratio had increased over time at most frequencies (Supplementary Figure 3). This increase is thought to represent an increase in central neural activity following an insult to the peripheral auditory system (Dehmel et al., 2012; Gu et al., 2012; Lowe and Walton, 2015). A repeated measures mixed effects analyses showed a significant effect of treatment at 32 kHz [*F*(1,195) = 7.068, *p* = 0.0011] for wave III/wave I ratios (Figure 6B). *Post hoc* tests showed that the control group was significantly different from the DMSO treated group at 6 weeks (*p* = 0.0029) and 10 weeks (*p* = 0.0030) at 32 kHz. Similarly, the ebselen treated group was significantly different from controls at week 6 (*p* = 0.0262) and week 10 (*p* = 0.0154) at 32 kHz. Importantly, ebselen co-treated mice were observed to have significantly lower ratios than DMSO treated animals at week 10 for both 16 kHz (*p* = 0.484) and 32 kHz (*p* = 0.480), where the maximum ratios were observed (Figure 6B). Since both the startle magnitude and the ABR wave III/I ratios increased following amikacin treatment, and ebselen treatment, alleviated this increase in both assessments, we decided to investigate if these data correlated in a meaningful way. A linear regression compared raw data for 90 dB startle magnitude to 16 kHz 40 dB SPL ABR wave III/I ratios at 4 epochs (Figure 6C). The vehicle group correlation increased until 10 weeks after treatment which was significant with *R*^2^ = 0.5781 (*p* = 0.0006) and then decreased again at the 14-week epoch. Interestingly, the ebselen group did not follow this pattern, as no significant correlations were observed at any epoch.

**FIGURE 7:**
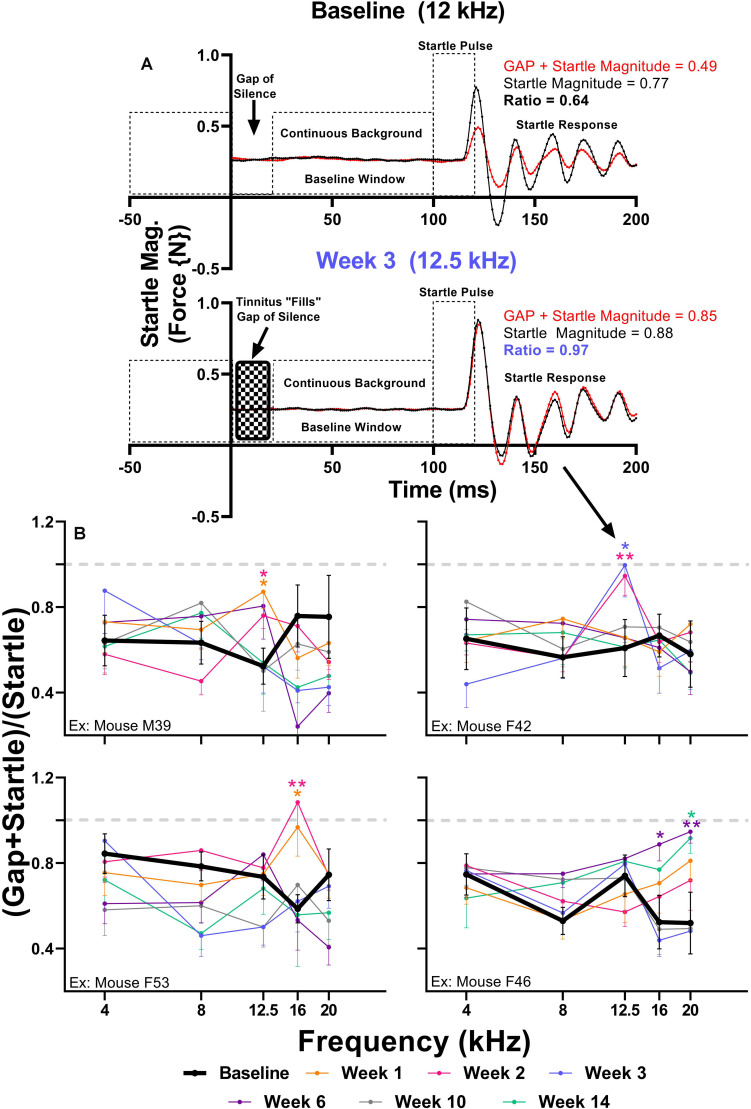
**(A)** Diagram of the GPIAS paradigm, including the stimuli used and the corresponding animal reactions. Representative GPIAS trial data from baseline and week 3 from an animal (mouse F42) which developed tinnitus at 12.5 kHz after amikacin treatment. The top panel (baseline) shows that a gap of silence embedded in a continuous background noise preceding a startle stimulus can reduce the startle response (red line) when compared to a situation when no gap is present (black line). In the bottom panel (week 3) the gap of silence is filled with tinnitus, resulting in diminished gap detection represented by a GPIAS ratio approaching 1.0. **(B)** GPIAS assessments in 4 (out of 17) mice with behavioral evidence of tinnitus. Tinnitus was defined as consistent and significant GPIAS deficit at 1 or 2 adjacent frequencies at weeks 1, 2, 3, 6, 10, or 14 when compared to baseline GPIAS performance. Data are represented by ratio means and standard errors. *Post hoc* significant differences between specific epochs (color coded) and baseline are indicated as follows: ^∗^*p* ≤ 0.05; ^∗∗^*p* ≤ 0.01. The dashed gray line (equaling a ratio of 1) represents no gap detection.

Brain hyperactivity has also been linked to behavioral evidence of tinnitus. To investigate if amikacin treatment leads to behavioral evidence of tinnitus development in mice, we used the GPIAS assessment across several epochs. When an animal perceives tinnitus, the internal noise of tinnitus occludes the gap of silence leading to gap detection ratios which approach 1.0 (Figure 7A). When evaluating gap detection prior to, and after amikacin treatment for individual animals, 4 of the 17 mice (23.5%) developed behavioral signs of tinnitus (Figure 7B). Mixed-effect analysis did not demonstrate a significant effect of epoch for mouse M39 [*F*(5.088,331.6) = 0.8346, *p* = 0.5275] or mouse F42 [*F*(5.25,315.0) = 0.5511, *p* = 0.7460], however, *post hoc* tests show a consistent deficit at 12.5 kHz at week 1 (*p* = 0.0312) and week 2 (*p* = 0.322) for Mouse M39 as well as week 2 (*p* = 0.0032) and week 3 (*p* = 0.0480) (Figure 7B). Mixed-effect analysis showed highly significant effect of epoch for mouse F53 [*F*(5.339,353.3) = 4.973, *p* = 0.0001] and mouse F46 [*F*(5.346,336.8) = 3.024, *p* = 0.0093] (Figure 7B). *Post hoc* tests revealed significant deficits at 16 kHz at weeks 1 (*p* = 0.0269) and week 2 (*p* = 0.0037) for mouse F53 and at 16 kHz at week 6 (*p* = 0.0324) and 20 kHz at week 6 (*p* = 0.0091) and week 14 (*p* = 0.0405) for mouse F46. Significant effects of amikacin treatment were not observed between DMSO and ebselen groups for gap detection deficits.

## Discussion

### Temporary Threshold Shifts in the Absence of Detectable Hair Cell, Ribbon Synapse, or Spiral Ganglion Loss

In an *in vivo* aminoglycoside mouse model, we found that amikacin can induce mild temporary ABR threshold shifts that fluctuate over a 3–4-month time period (Figure 2). These results are similar to the reported observation from other recent aminoglycoside experiments (Murillo-Cuesta et al., 2010; Ogier et al., 2020). The percentage of ears that showed clinically relevant hearing loss was minimal and concentrated at times closest to the amikacin treatment and gradually diminished to near baseline levels by week 14 (Figure 2 and Table 1; American Speech-Language-Hearing Association [ASHA], 1994; Gu et al., 2020). This minimal threshold shift was expected as mice have shown similar levels of resistance to AG-ototoxicity as humans, but more resistance than rats and guinea pigs. Such differences have been attributed to factors such as pharmacokinetics, bioavailability, and activation of the drug (Wu et al., 2001; Fernandes and Lin, 2014). Human AG studies have reported a hearing loss prevalence between 0 and 47%, however, these estimates are highly dependent on the specific hearing loss criteria and specific inclusion/exclusion criteria for the study population (Garinis et al., 2017). This suggests that CBA/CaJ mice could be considered an appropriate animal model for AG-cochleotoxicity as we found hearing loss rates of 18–36% using strict criteria modeled from ASHA guidelines (Table 1; American Speech-Language-Hearing Association [ASHA], 1994). However, our rates may have been higher if more ABR frequencies were tested, which needs to be examined in future studies. In general, most mice strains have shown low levels of cochleotoxic change that correspond well to human studies (Ogier et al., 2020). However, most of animal studies utilized one dose of AG, which does not correlate to the cumulative effects of AG-induced cochleotoxicity seen in CF patients receiving AG treatment throughout life (Garinis et al., 2017). Therefore, future animal studies should focus on cumulative AG-related cochleotoxicity.

Interestingly, our data suggest that ABR threshold shifts were not due to the loss of hair cells (Figure 3). One recent study also showed minor threshold shifts with minimal hair cell loss following treatment with both amikacin and tobramycin in mice. This is also a common finding in the literature when clinically comparable doses of AGs are used (Ogier et al., 2020). We did not observe any detectable loss of SGNs (Supplementary Figure 1), or ribbon synapses (Figure 4). However, future studies should use more detailed methodologies to identify and quantify small, and possibly temporary changes to these critical sensory cells. While this study was not able to detect obvious changes in SGNs or ribbon synapses, previous studies have shown small temporary changes in these specific sensory structures following exposure to AGs. Recent evidence has suggested that ribbon synapses degrade following low doses of AG treatment corresponding to declines in hearing sensitivity in the absence of hair cell or SGN loss (Liu et al., 2013). Interestingly, a follow-up study found that these synapses partially repair after the cessation of AG treatment (Liu et al., 2015). Recent investigations suggest this phenomenon can be explained by AMPA and NMDA receptor reorganization following glutamate excitotoxicity (Hong et al., 2018). It was shown that partially preventing such glutamate hyperexcitability from both noise and amikacin via NMDA antagonists could prevent ribbon synapse damage in mice and guinea pigs (Duan et al., 2000; Hong et al., 2018). Because glutamate excitotoxicity is mediated by reactive oxygen species (Mark et al., 2001), it is likely that AG-induced damage to cochlear structures is caused by inflammation (Kamogashira et al., 2015). A more in-depth study should be conducted to investigate if ribbon synapse reformation/plasticity is correlated with the timeline of behavioral symptoms of hyperacusis or tinnitus. It is also possible that thickening of the stria vascularis and subsequent changes in endocochlear potentials could explain the changes in threshold shifts (Hirose et al., 2014), and this should be further studied.

Ebselen, a mimic and inducer of GPx, has demonstrated the ability to ameliorate AG-induced threshold shifts by neutralizing ROS (Figure 2; Gu et al., 2020), similar to other antioxidant drugs but at significantly lower oral doses (Mukherjea et al., 2015; Fox et al., 2016; Hammill and Campbell, 2018). These findings were not surprising as it is known that ebselen has shown robust protection against cisplatin- and noise-induced hearing loss in animals and humans at low oral doses (Lynch et al., 2005; Kil et al., 2007, 2017). As no obvious histological damage was observed in this study, ebselen was not shown to have a protective effect on the cochlea (Figures 3, 4 and Supplementary Figure 1). However, the plasticity following AG treatment might also be explained by an inflammatory dynamic between macrophages and SGNs, that was recently demonstrated in a model of noise-induced hearing loss (Kaur et al., 2019). A more detailed timeline of peripheral vs. central AG-induced dysfunctions should also be elaborated, as it is known that some drugs stay in the inner ear for long periods of time, causing a state of continued inflammation (Breglio et al., 2017). A limitation of this study is that we did not investigate cochlear or brain inflammation, but previous studies have shown that AGs do lead to transient inflammatory states (Jiang et al., 2017). Additionally, AGs may cross the blood–labyrinth (Kalinec et al., 2017) and blood–brain barriers in children (Gaillard et al., 1995) and in elderly adults (Mattappalil and Mergenhagen, 2014). Thus, future animal studies might refine the doses and compare different AGs to further detail the otoprotective effects of ebselen on ototoxin-induced cochlear inflammation.

### Amikacin Leads to Behavioral Evidence of Hyperacusis and Tinnitus Which Correlates to Increased Brainstem Activity

The ASR methodology has been utilized to assess behavioral evidence of noise-induced tinnitus (Turner et al., 2006; Longenecker and Galazyuk, 2011; Middleton et al., 2011; Dehmel et al., 2012) and hyperacusis (Sun et al., 2012; Chen et al., 2013; Hickox and Liberman, 2014) in many animal models. It is hypothesized that tinnitus and hyperacusis, in both animals and humans, are caused by central maladaptation following damage to the auditory periphery (Roberts et al., 2010; Langguth et al., 2013; Auerbach et al., 2014; Pienkowski et al., 2014). Here, we report the first evidence that AG treatment can lead to similar centrally based symptoms of maladaptive plasticity (Figures 6, 7). Hyperacusis was observed immediately after the start of AG treatment and persisted until week 6 in the ebselen treated group, and week 14 in the vehicle group (Figure 6A). The magnitude and consistency of the I/O startle response magnitudes were surprising considering that startle magnitude usually habituates over repeated testing sessions (Figures 5C,E; Davis, 1984; Longenecker et al., 2018). Since 14 out of the 17 mice demonstrated greatly exaggerated startle responses, behavioral evidence of hyperacusis could be a common outcome of AG treatment. Interestingly, gap detection deficits thought to represent behavioral evidence of tinnitus (Turner et al., 2006), were less common than behavioral evidence of hyperacusis, with only 4 of the 17 (24%) mice demonstrating statistically significant frequency-specific deficits at multiple timepoints (Figure 7B). This is not unlike the 19.4% tinnitus rate found in a human study examining the prevalence of new tinnitus symptoms after ototoxic antibiotics like amikacin (Dille et al., 2010). Significant gap detection deficits were observed in the acute phase (during or immediately following treatment) in 3 out of the 4 mice (Figure 7B). This pattern of tinnitus was closely associated with behavioral evidence of hyperacusis, which is not surprising the high rate of clinical comorbidity (Hébert et al., 2013; Knipper et al., 2013; Schecklmann et al., 2014; Roberts and Salvi, 2019). While this study showed that hyperacusis can be prevented to some degree, future studies will investigate if tinnitus/hyperacusis can be ameliorated when ebselen is given after these symptoms have already been developed, as has been shown with noise- and drug-induced tinnitus models (Brozoski et al., 2007; Lobarinas et al., 2011; Galazyuk et al., 2019).

We found that these behavioral manifestations of auditory dysfunction were correlated with ABR wave III/I amplitude ratio in the vehicle treated group (Figures 6B,C). ABR wave I is thought to originate from the SGNs within the cochlea, while wave III is thought to derive from neurons of the ventral cochlear nucleus/superior olive within the brainstem (Melcher and Kiang, 1996). We found an increase in the wave III/I ratio which suggests an increase in central activity following AG treatment (Figure 6B). This phenomenon has also been observed in noise-induced hearing loss and salicylate-induced tinnitus animal models (Dehmel et al., 2012; Gu et al., 2012; Lowe and Walton, 2015). This evidence suggests that the amikacin treatment model could be a good candidate for studying temporary central gain increases due to peripheral inflammation. Importantly, the timeline of deficits for the amikacin/DMSO group was correlated until the week 10 epoch, with the ABR deficits developing slower than the behavioral deficits (Figures 2, 6). While the amikacin/ebselen group demonstrated increased thresholds, and startle magnitudes, it was not to the same level or duration as the DMSO group. This is particularly intriguing in the absence of significant peripheral damage and may only be temporary in animal models with clinically relevant dosing (Figures 3, 4; Gu et al., 2010; Sheldrake et al., 2015). A more detailed study would include a complete experimental analysis of ribbon synapses, perhaps at varying AG dosing levels to tease apart this proposed mechanism, and at several epochs following AG administration. However, it is uncertain if the behavioral manifestations of hyperacusis/tinnitus seen in this study are caused by peripheral or central inflammation (Fuentes-Santamaría et al., 2017; Wang et al., 2019). We hypothesize that AGs lead to inflammation of the auditory nerve fibers resulting in hyperactivity, which may ascend through the central auditory pathway. However, AGs may also cross the blood–brain barrier and directly cause the behavioral auditory dysfunctions seen in these series of experiments. Interestingly, some evidence has shown that anti-inflammatory drugs like melatonin may reduce tinnitus clinically (Reiter et al., 2011). Future studies should utilize single and/or multi-unit electrophysiological recordings to identify such a cellular mechanism.

### Limitations of the ASR and ABR Methodology

Many of the basic assumptions of the GPIAS methodology for tinnitus assessment have been questioned in human studies. Such studies have shown that participants with tinnitus are able to detect gaps of silence in a background noise (Campolo et al., 2013; Boyen et al., 2015), thus invalidating the original hypothesis developed in rats (Turner et al., 2006). These results may be explained by a recent study which found that tinnitus is perceived as separate from external sounds, thus not interfering with gap detection (Zeng et al., 2020). However, some reports have suggested that gap detection differences can be identified in patients with tinnitus when assessed with cortical evoked potentials (Mahmoudian et al., 2013, 2015; Paul et al., 2018). GPIAS has also been questioned in animals, as it was shown that when operant tasks requiring conscious perception are used to assesses salicylate-induced tinnitus, rats did not demonstrate gap detection deficits like the ones observed in this and other ASR based studies (Galazyuk and Hébert, 2015; Radziwon et al., 2015; Figure 7). Hearing loss has been shown to be a limitation for ASR-based gap detection tests (Lobarinas et al., 2013), but in this study AG-induced threshold shifts were minimal (Figure 2). ASR evaluations of enhanced hearing sensitivity have been less scrutinized thus far, but it is important to note that hyperacusis is thought involve maladaptive changes to large networks in the brain (Auerbach et al., 2014). The ASR and ABR used for hearing assessments both evaluate circuitry thought to be limited the brainstem (Melcher and Kiang, 1996; Koch, 1999), and do not encompass the vast subcortical and cortical auditory/non-auditory network that is thought to be engaged in sound perception or perceptual disorders like tinnitus and hyperacusis. However, maladaptive changes in brainstem nuclei, like the ones presented in this study, may lead to upstream perceptional consequences of hyperacusis and tinnitus (Knipper et al., 2013; Wu et al., 2016). Future animal studies using AGs should employ operant tasks for assessing gap detection (Radziwon et al., 2015) and hyperacusis (Manohar et al., 2017) in combination with ASR-based assessments to compare conscious and unconscious assessments.

### Clinical Relevance and Future Studies

Results from this animal study show that auditory dysfunctions such as hyperacusis and tinnitus may accompany or be independent of the significant hearing loss associated with AG treatment. While hearing loss is the most significant factor for tinnitus and hyperacusis (Knipper et al., 2013), several studies have reported that patients have tinnitus and hyperacusis in the absence of clinically significant threshold shifts (Schmuziger et al., 2006; Job et al., 2007; Gu et al., 2010; Langers et al., 2012; Sheldrake et al., 2015). Recent work in animals has also shown this to be true (Longenecker and Galazyuk, 2011, 2016; Dehmel et al., 2012; Turner et al., 2012). Although we did not observe significant synapse degradation/loss, we did observe changes in ABR wave III/I ratios indicating physiological changes between the peripheral and central auditory system (Dehmel et al., 2012; Gu et al., 2012; Lowe and Walton, 2015), which may be explained by “synaptopathy/hidden hearing loss,” or damage to the peripheral afferent system in the absence of significant hair cell loss (Schaette and McAlpine, 2011; Kujawa and Liberman, 2015). Growing evidence suggests that SGNs or their synapses are preferentially targeted by AGs (Liu et al., 2013; Hong et al., 2018) which has also been shown to be true in histological analysis from human cadavers (Sone et al., 1998; Pauna et al., 2017). Importantly, mechanistic understandings of the peripheral and central issues related to AG treatment should be elucidated further.

Clinical evaluation of AG-induced synaptopathy and/or hidden hearing loss should be further investigated with behavioral assessments like speech-in-noise tests (Le Prell, 2019), or physiological tests like ABR or the middle-ear-muscle reflex (Bharadwaj et al., 2019; Guest et al., 2019). Since hyperacusis and tinnitus may become chronic bothersome conditions, further work is necessary to investigate their incidence and severity (Baguley and Andersson, 2007; Pienkowski et al., 2014; Fackrell et al., 2017). A recent study found that LDLs were highly correlated to the ASR which suggests that the ASR could be a valid measure for assessing hyperacusis in humans and animals (Knudson and Melcher, 2016). However, this study and others like it found that self-reported (SLTQ) hyperacusis was not correlated to LDLs. The authors reasoned that these results could be explained by two assessments trying to explore the relationship between different types of sounds (laboratory vs. sounds experienced in everyday life). Alternatively, it could be explained that perceptual deficits (reduced LDL) can coexist with differences in awareness of the bothersome nature of sounds, or hyperacusis. Exploratory studies using LDL assessments and self-report hyperacusis questionnaires should be conducted on AG receiving patients to investigate if hyperacusis contributes to overall auditory dysfunction associated with single and multi-course AG treatment.

## Data Availability Statement

The raw data supporting the conclusions of this article will be made available by the authors, without undue reservation.

## Ethics Statement

The animal study was reviewed and approved by Institutional Animal Care and Use Committee at Sound Pharmaceuticals, Inc.

## Author Contributions

RL, RG, and JK designed the research. RL, RG, and JH performed the research. RL and RG analyzed the data. RL and JK wrote the manuscript. All authors contributed to the article and approved the submitted version.

## Conflict of Interest

The authors disclose that they are employed by Sound Pharmaceuticals and have stock ownership in the Company. The authors declare that this study received funding from Sound Pharmaceuticals, Inc. The funder had the following involvement with the study: provided full financial support for this study.

## Abbreviations

ABR: auditory brainstem response
AG: aminoglycosides
ANOVA: analysis of variance
ASR: acoustic startle reflex
CF: cystic fibrosis
CMD: center of mass displacement
DMSO: dimethylsulfoxide
GAP: trial with gap preceding startle
SO: trial with startle only
GPIAS: gap-induced prepulse inhibition of the acoustic startle reflex
IHC: inner hair cell
IO: input/output
kHz: kilohertz
LDL: loudness discomfort level
OHC: outer hair cell
SGN: spiral ganglion neurons
TB: tuberculosis.
