# Supplementary material for: A Novel Mouse Model of Aminoglycoside-Induced Hyperacusis and Tinnitus
Source: Front Neurosci. 2020 Sep 18;14:561185. doi: 10.3389/fnins.2020.561185 (PMC7530258; doi:10.3389/fnins.2020.561185)
Supplement: Supplementary file 1 [file Data_Sheet_1.docx]

Supplementary Material

**Supplemental Figure/Table Legends:**

**Supplemental Figure 1.** Representative mid-modiolar cross sections comparing spiral ganglion neurons and hair cells between dosing groups (A - C: amikacin, D - F: amikacin/ebselen). No observable evidence of amikacin-induced spiral ganglion neuron loss or hair cell loss was seen in the apical turn (A, D), the middle turn (B, E), or the basal turn (C, F) for either group. Scale bar = 20 mM.

**Supplemental Figure 2.** Averaged ABR wave I and III amplitudes from 40 dB SPL stimuli comparing dosing groups at baseline and different timepoints after AG treatment. A - D: Wave I amplitudes at 4, 8, 16, and 32 kHz stimuli. E-H: Wave III amplitudes at 4, 8, 16, and 32 kHz stimuli. Data are represented by amplitude mean (peak to peak voltage) and standard errors.

**Supplemental Figure 3.** Averaged ABR Wave III/I ratios (%) from 40 dB SPL stimuli comparing groups at baseline and different timepoints after AG treatment. A - D: Ratios from 4, 8, 16, and 32 kHz stimuli. Date are represented by ratio means and standard errors. Significant differences between testing groups are indicated as follows: #, between control and DMSO treated animals, # (gray), between control and ebselen treated animals, *, between DMSO and ebselen treated animals.

**Supplemental Table 1.** Post hoc significance levels and p-values for figure 6A. Data presented sound intensities above 70 dB SPL at the 6- and 10-week epochs. Significant differences between testing groups are indicated as follows: #, between control (C) and DMSO (D) treated animals; # (gray), between control and ebselen (E) treated animals; *, between DMSO and ebselen treated animals.

**Supplemental Figures:**

**Supplemental Figure 1:**


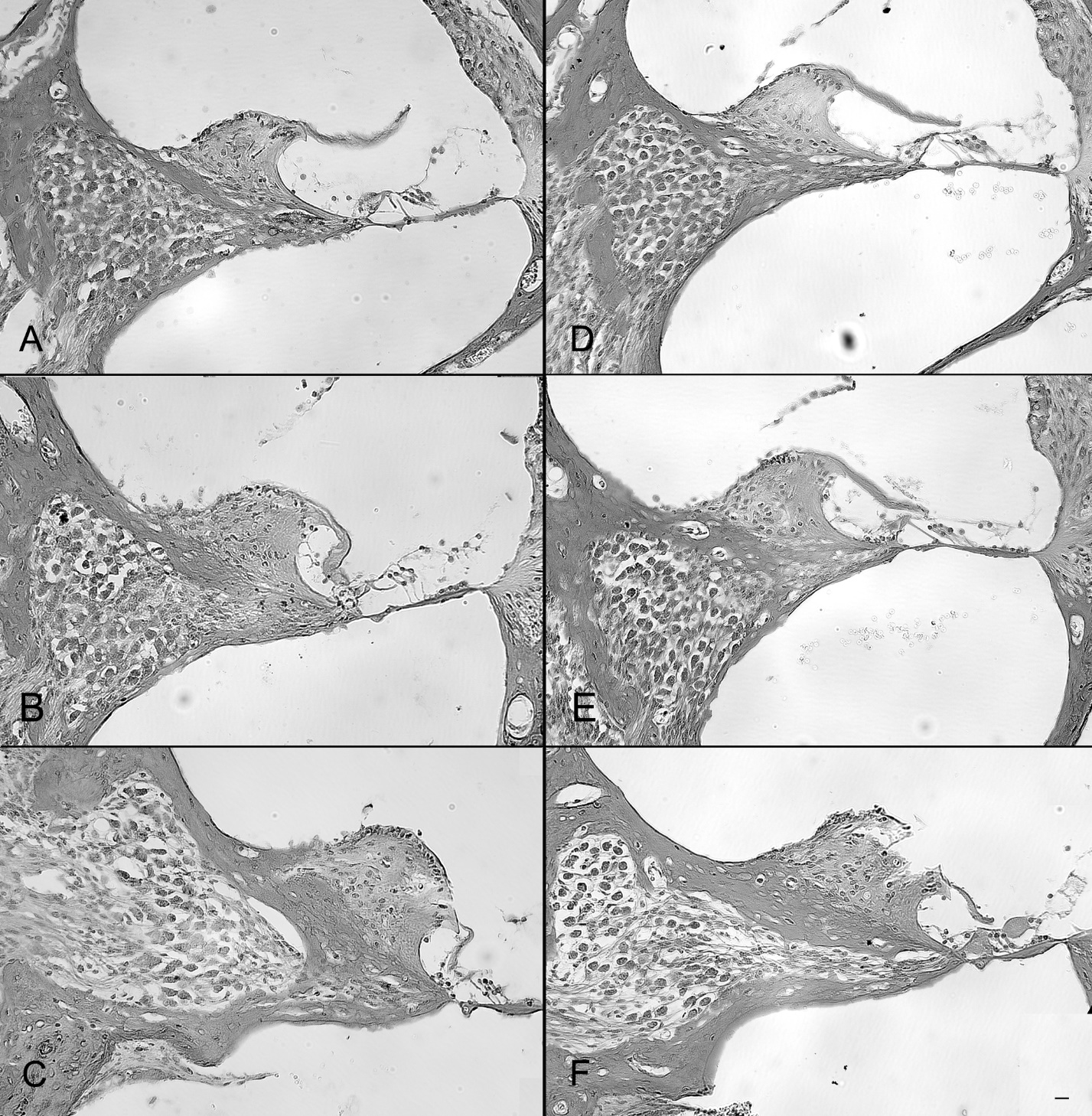


**Supplemental Figure 2:**


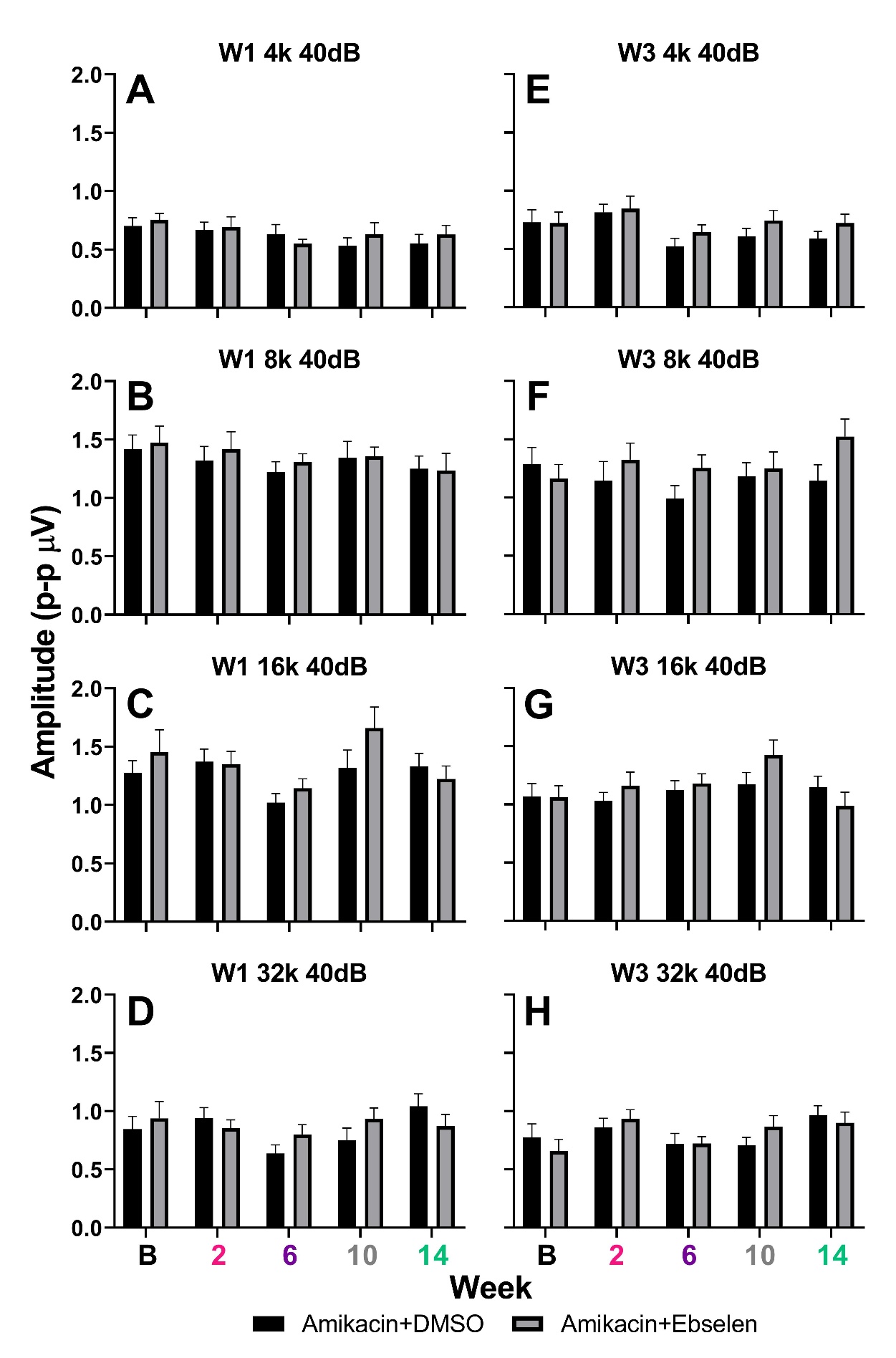


**Supplemental Figure 3:**

**
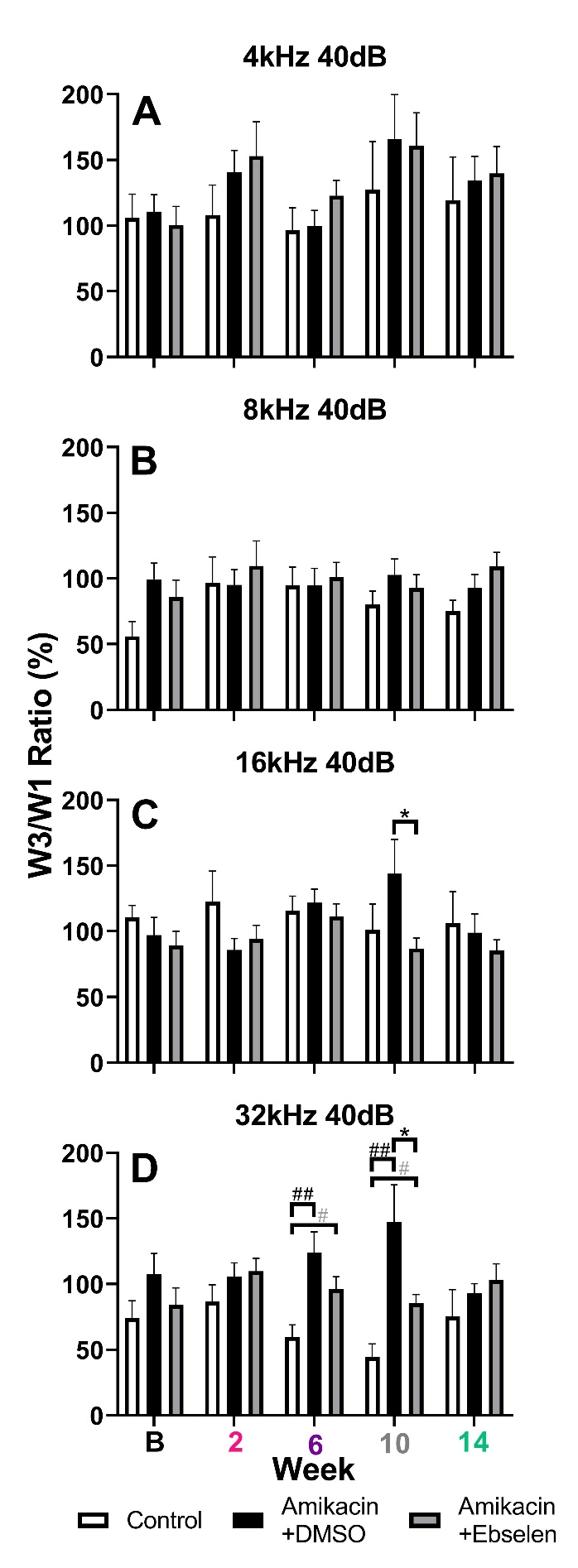
**

**Supplemental Table 1:**
